# Profiling oral and digital lesions in sheep in Ireland

**DOI:** 10.1186/s13620-015-0055-0

**Published:** 2015-12-16

**Authors:** William G. FitzGerald, Joseph P. Cassidy, Bryan K. Markey, Michael L. Doherty

**Affiliations:** Department of Agriculture, Food and Marine, Regional Veterinary Laboratory, Leggetsrath, Hebron Road, Kilkenny, Ireland; School of Veterinary Medicine, University College Dublin, Belfield, Dublin 4, Ireland

**Keywords:** Foot and mouth disease, Sheep, Oral lesions, Digital lesions, Differential diagnoses

## Abstract

**Background:**

During the FMD outbreak in Ireland and the UK in 2001, there was significant uncertainty amongstveterinary practitioners and government veterinary inspectors surrounding the clinical diagnosis of FMD insheep. This situation was complicated by reports of idiopathic oral ulcers that closely resembled FMD ongross appearance which at that time were referred to as ovine mouth and gum obscure disease.

**Methods:**

A field and abattoir study was carried out to determine the frequency, appearance and significance of oraland digital lesions in sheep in Ireland. A total of 3, 263 sheep were examined in 22 flocks, including 1, 969lambs and 1, 294 adults. A further 2,403 animals were examined by abattoir inspections. Animals bearing lesions of interest were identified, samples of the lesions were taken and subsequently examined by bacteriology, electron microscopy, serology, immunohistochemistry and histopathology.

**Results:**

Forty four oral and 20 digital lesions were identified and characterised. Oral lesions were recorded mostfrequently in lambs, where the most common cause was orf virus infection. The majority of the oral lesions recorded in the adults was idiopathic and consistent with a diagnosis of idiopathic oral ulceration. A variety of digital lesions was observed, consistent with scald, foot-rot and contagious ovine digital dermatitis (CODD). All of the animals with lesions were seronegative to FMD virus (FMDV).

**Conclusions:**

There was no difficulty in differentiating these lesions from those caused by FMDV on the basis of flockhistory and careful clinical examination.

**Electronic supplementary material:**

The online version of this article (doi:10.1186/s13620-015-0055-0) contains supplementary material, which is available to authorized users.

## Background

Foot-and-mouth disease (FMD) is a highly contagious viral disease of ungulates that has a very significant economic impact [1]: the cost of the FMD outbreak in the UK in 2001 was approximately £3.1 billion [2]. Foot-and-mouth disease causes vesicular lesions on the feet, particularly at the coronary band, in the interdigital cleft and at the heel bulb [3]. FMD can also produce, though less commonly in sheep, vesicles in the oral cavity, with predilection sites on the dental pad and on the dorsal tongue [3]. Oral lesions similar to those caused by FMD can be caused by diseases such as bluetongue [4], idiopathic oral ulceration [5, 6], vesicular stomatitis (in cattle) [7,8] and contagious pustular dermatitis (orf) [9].

During the FMD outbreak in Ireland and the UK in 2001, there was significant uncertainty amongst veterinary practitioners and government veterinary inspectors surrounding the clinical diagnosis of FMD in sheep. This situation was complicated by reports of idiopathic oral ulcers that closely resembled FMD on gross appearance, then referred to as ovine mouth and gum obscure disease [6].

This diagnostic uncertainty highlighted the lack of published information surrounding the gross appearance and prevalence of lesions seen in the FMD-free sheep population. The objectives of this study were to address this deficiency by systematically identifying, describing and investigating the cause of the range of background oral and digital lesions in FMD-free sheep in Ireland. It was anticipated that the information gleaned from this work would greatly assist veterinary practitioners and inspectors in reaching a more informed diagnosis when confronted with such lesions ‘in the field’.

## Materials and methods

### Abattoir study

An abattoir study was undertaken to mimic the conditions under which private veterinary practitioners, in their role as temporary veterinary inspectors acting on behalf of the Department of Agriculture, Food and the Marine, were experiencing pressure in diagnosing particlularly oral lesions of unknown aetiology.

Three abattoirs were chosen based on their high throughput of sheep and their proximity to University College Dublin. The abattoirs slaughtered sheep from all over the Republic of Irelend but the majority of their intake was from the province of Leinster. A total of 2,403 sheep of all ages were examined for oral and digital lesions in the lairages of these facilities between November 2002 and November 2003. Sheep were examined at random and those displaying lesions *ante mortem* were identified and all four feet as well as the heads were collected *post mortem* for sampling and study.

### Flock study

Twenty-two commercial flocks were sourced ranging in size from 122–600 ewes. Of the 22, three of the flocks were hill flocks, 18 were lowland and one was mixed. The majority of the flocks were in Leinster, in counties Meath, Wicklow and Carlow. A small number of flocks in Cavan and Monaghan were also included. The most common breeds of ewe kept on the 22 farms were Suffolk, Cheviot and Texel, while the most common ram breeds were Suffolk, Charollais and Texel. Each flock was visited on three or four occasions during the investigation. Flocks were selected based mainly on the reality of sheep farmers who were willing to cooperate with the study and the significant inconvenience that the visits would cause. Flocks were recruited with the good will of farmers with the support of Teagasc, the Irish Agricultural advisory service. Visits were designed to inspect sheep at different stages of the production cycle: adults while housed prior to lambing, lambs at six to ten weeks of age, lambs on pasture ready for slaughter, adults at pasture. During each visit a sample of 10-20 % of the flock was chosen at random by gathering the sheep and after counting the number of sheep, 10-20 % was calculated. Sheep were selected by walking through the group and every 5–10 sheep marking one with spray. A count was kept of marked sheep. Once the required total was marked, the marked sheep were separated and examined. Each animal within that sample group was examined for evidence of peri-oral, oral and digital lesions. The number of sheep examined was dictated by the size of the flock, the handling facilities and the willingness of the flock owners. In larger flocks (>250 ewes) 10-15 % of the flock was examined, while in smaller flocks (<250 ewes), 20 % of the flock was examined; a total of 3,263 sheep were examined for the presence of lesions. Examination consisted of individually casting sheep and examining the peri-oral region of each animal visually and by palpation. Subsequently, the mouth was opened manually and with the aid of a mouth gag as appropriate, the mucosae, gingivae and tongue were visually inspected. All four feet were examined, with emphasis on the interdigital space, horn and coronary band. All sheep exhibiting lesions were subjected to a full clinical examination including the recording of rectal temperature and sampling procedure as described below.

### Samples

Samples taken from each lesion consisted of a swab for bacteriological examination, a tissue sample for histopathological examination and a tissue sample for electron microscopy (EM). The swab was placed in Amies transport medium (Medical Supply Company, Ireland) for transport to the laboratory. Following local regional analgesia using 2 % lignocaine (Norocaine, Norbrook, United Kingdom), sedation using 2 % xylazine (Chanazine, Chanelle, Ireland) or general anaesthesia using ketamine 10 % (Narketan, Vetoquinol, France) as required, a biopsy was taken from each lesion using an 8 mm circular biopsy punch (Stiefel Laboratories, Ireland). Half of the biopsy was placed in a universal container and stored at −70 °C for examination by electron microscopy and the other half was placed in a universal container containing 10 % formalin for histopathological examination. In addition, a clotted blood sample was taken from each sheep with a lesion by jugular venipuncture.

### Bacteriology

Samples were incubated at 37 °C under three different atmospheric conditions: aerobic, microaerophilic and anaerobic. Samples for aerobic incubation were cultured on McConkey agar and blood agar containing 5 % defibrinated sheep blood with colistin and nalidixic acid (CNA, LabM, Bury) [10]. Microaerophilic conditions, were maintained using Gaspak® system (BBL, Becton Dickinson, Maryland, USA) and samples inoculated onto blood agar plates. Samples were also inoculated onto fastidious anaerobic agar [10–12]. Isolates were identified using Gram stain, catalase and oxidase tests and the use of API® strips including API Staph®, API 20 Strep®, API 20NE®, API 20 E® and rapid PIA 32A® (Biomerieux, UK).

### Electron microscopy

Samples were prepared and examined for the presence of viral particles as described by Harkness *et al*. [13].

### Histopathological examination

Formalin-fixed samples were embedded in paraffin wax using standard procedures, cut into 3-5 μm thick sections, mounted on slides and stained with haematoxylin and eosin (HE) [14].

### Immunohistochemical staining for orf virus antigen

Tissue sections were selected for confirmatory immunohistochemical labeling on the basis of histopathological and electron microscopy results. Unstained tissue sections were placed on ‘sticky’ slides and labelled with a murine monoclonal antibody 2E5 [15] specific for an envelope protein on the orf virus [16]. This examination was undertaken by staff at the Moredun Research Institute under the supervision of Dr. Peter Nettleton.

### Serological testing for foot-and-mouth disease virus (FMDV)

Collected sera were stored at −70 °C and the sera were batch-tested by liquid phase blocking (LPB) ELISA to detect antibodies to serotype O of FMDV [17]. The LPB-ELISA was performed according to protocols provided by the Institute for Animal Health, Pirbright, UK [18].

## Results

Sixty four individual animal lesions were recorded during the entire study, 52 (81 %) in the flock study and 12 (19 %) in the abattoir study, respectively. For simplicity of classification, lesions located within the oral cavity and around the oral cavity (peri-oral cavity) were classified as oral lesions.

### Abattoir study

A total of 2,403 sheep were examined during the abattoir study and 12 (0.5 %) oral lesions were identified, these were investigated in detail. Lesions found during this study were most commonly located on the dental pad (8/12, 67 %).

### Flock study

In the course of the flock study, 3,263 sheep were examined comprising 1,264 adults and 1,969 lambs. Of the 3,263 sheep, 32 (1 %) sheep displayed oral lesions, none exhibited pyrexia or any other significant clinical abnormalities. Of the 3,263 sheep, 20 (0.6 %) sheep bore digital lesions, six were overtly lame on one limb, three of which were non-weight bearing.

Twelve lesions (0.9 %) were detected amongst the surveyed adult population, seven (0.5 %) oral lesions and 5 (0.4 %) digital lesions. During the flock study, numerous lame sheep were detected but due to the limited resources available, the most representative digital lesions were sampled.

Among the lambs, 40 (2 %) animals with lesions were detected comprising 25 (1.3 %) oral lesions and 15 (0.7 %) digital lesions. Of the 25 oral lesions, 12 (48 %) were confirmed as orf by EM, immunohistochemistry and histopathology.

In total, 64 lesions were described, there were 44 oral lesions (25 erosive/ulcerative and 19 exophytic) and 20 digital (13 erosive/ulcerative and 7 exophytic).

### Histopathological examination

Oral and digital lesions from both studies were divided on the basis of morphological characteristics into two categories, erosive/ulcerative and exophytic lesions. Descriptions of the size, shape, anatomical location and number of lesions are shown in Tables 1, 2, 3 and 4. The histopathological features of the lesions in each of the four categories are shown in Tables 5 and 6. Erosive/ulcerative lesions were defined as those where there was partial or complete focal loss of the surface epithelium [19]. Exophytic lesions were defined as lesions which were growing/extending outwards from the skin/mucosal surface [20].

**Table 1:** Description of the size, shape, number and specific anatomical location of the lesions encountered during the study

Size				
	≤0.5 cm	0.6 – 1.0 cm	1.1 – 2.0 cm	>2.0 cm
Digital Exophytic	0	1	3	3
Digital Erosive/Ulcerative	0	1	1	11
Oral Exophytic	5	6	1	7
Oral Erosive/Ulcerative	7	10	3	5

**Table 2:** Description of the size, shape, number and specific anatomical location of the lesions encountered during the study

Shape								
	Irreg.	Circ.	Semicirc.	Hemispherical	Elliptical	Linear	Kidney	Rect.
Digit Exo	4	0	0	1	0	0	0	2
Digit Ulcer	9	0	0	2	1	0	0	1
Oral Exo	8	1	0	9	0	0	1	0
Oral Ulcer	5	6	1	1	4	8	0	0

Key: Irreg. – Irregular; Circ. – Cicrular; Semicirc. – Semicircular; Rect. - Rectangular

**Table 3:** Description of the size, shape, number and specific anatomical location of the lesions encountered during the study

Location - digit					
	Cleats	Interdigital	Sole	Coronary band	Foot
Digital Exophytic	1	4	1	1	0
Digital Erosive/Ulcerative	5	4	1	2	1

**Table 4:** Description of the size, shape, number and specific anatomical location of the lesions encountered during the study

Location - oral						
	DentalPad	Lip	Nares	Mucosa	Skin	Mandible
Oral Exo	8	8	3	0	0	0
Oral Ulcer	9	7	1	6	1	1

**Table 5:** Description of the histopathological features of the oral lesions identified indicating the frequency with which each feature was identified in each lesion category.

Feature	Oral Erosive/Ulcerative (*n* = 25)	Oral exophytic (*n* =19)
Congestion and perivascular cuffing of dermal vessels (sub-categories were identified depending upon the predominant leucocyte type).	• Neutrophils (6 of 21)	• Neutrophils (1 of 18)
	• Lymphocytes (6 of 21)	• Lymphocytes (4 of 18)
	• Macrophages (1 of 21)	• Plasmacytes (1 of 18)
	• Plasmacytes (2 of 21)	• Mixed leucocyte population (12 of 18)
	• Mixed leucocyte population (6 of 21)	
Degeneration of epithelial cells /keratinocytes		• Vacuolar degeneration (12 of 13)
		• Reticular degeneration (10 of 13)
		• Ballooning degeneration (4 of 13)
Dermal haemorrhage	1	1
Epidermal/Mucosal erosion	6	4
Epidermal/Mucosal hyperplasia	14	15
Epidermal/Mucosal ulceration	10	2
Epidermal micropustules	4	10
Intra-lesional bacteria	6	1
Serocellular crust formation	6	7
Surface fibrinous exudate	5	0
Thrombosis of dermal vessels	0	3

**Table 6:** Description of the histopathological features of the digital lesions identified indicating the frequency with which each feature was identified in each lesion’s category

Feature	Digital Erosive/Ulcerative (*n* = 13)	Digital Exophytic (*n* = 7)
Congestion and perivascular cuffing of dermal vessels (sub-categories were identified depending upon the predominant leucocyte type).	• Neutrophils (4 of 11)	
	• Lymphocytes (1 of 11)	
	• Mixed leucocyte population (6 of 11)	• Mixed leucocyte population (6 of 11)
Degeneration of epithelial cells /keratinocytes	• Vacuolar degeneration (2 of 3)	• Vacuolar degeneration (4 of 4)
	• Reticular degeneration (1 of 3)	• Reticular degeneration (3 of 4)
Dermal fibroplasia	0	2
Epidermal erosion	5	0
Epidermal hyperplasia	6	5
Epidermal micropustule	6	6
Intra-lesional bacteria	5	2
Serocellular crust formation	1	4
Thrombosis of dermal vessels	1	2

### Oral Erosive/Ulcerative lesions

**Fig. 1:**
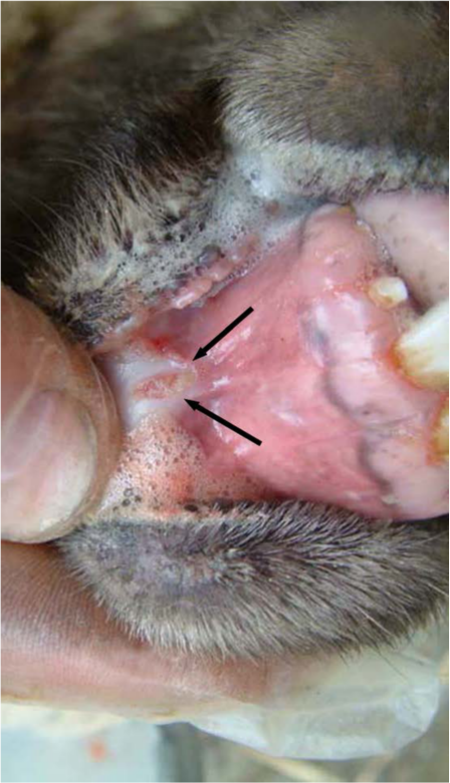
Photograph illustrating a, well demarcated, elliptical ulcer (black arrows), approximately 5 mm in diameter with a red centre on the lingual aspect of the lower lip, along the midline. The periphery of the lesion is blanched due to digital pressure. This lesion was observed in an 18 month-old female sheep grazing on very tightly grazed pasture. Detailed examination failed to detect the presence of pathogenic organisms. This combined with the location of the lesion and the grazing history suggests that this lesion was likely traumatic in origin

**Fig. 2:**
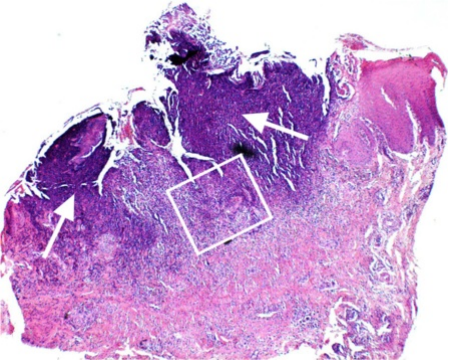
Photomicrograph, illustrating a section of the ulcer shown in Fig. 1. There is extensive loss of buccal epithelium with a dense infiltrate of inflammatory cells denoted by the intensely basophilically staining area (white arrows) (HE 4x). The area within the white box is shown magnified in Fig. 3

**Fig. 3:**
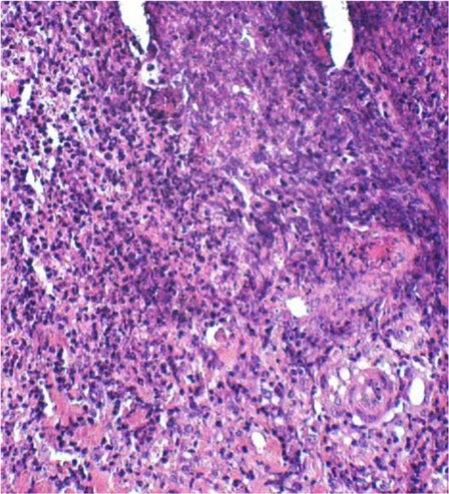
Magnified photomicrograph of the demarcated portion of Fig. 2, illustrating an area of predominantly neutrophilic inflammatory cell infiltrate (HE 20x)

**Fig. 4:**
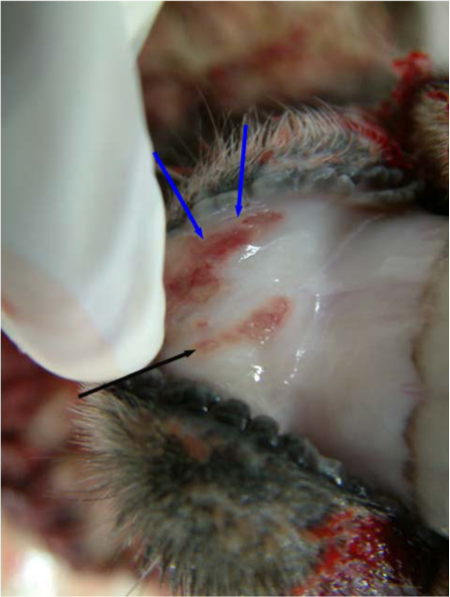
Photograph of two individual ulcers located on the lingual aspect of the lower lip of a ewe (photograph taken *post mortem*). The larger of the two ulcers is L-shaped (blue arrows) with a red core and yellow periphery. The smaller of the two ulcers is linear in shape with a red core and yellow periphery (black arrow)

**Fig. 5:**
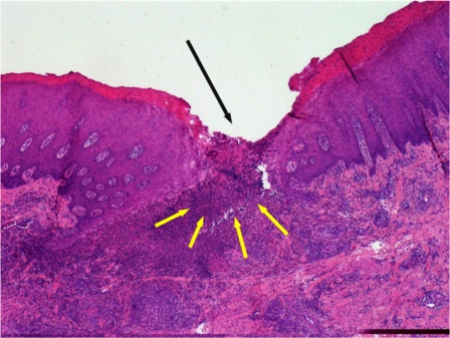
Photomicrograph of a section, taken from the L-shaped ulcer, highlighted by blue arrows, in Fig. 4. There is epidermal hyperplasia with a localised area of epidermal necrosis (black arrow). There is extensive inflammatory cell infiltrate extending from the ulcer (yellow arrows)

Twenty-five oral lesions from both studies were included in the erosive/ulcerative category (Figs. 1, 2, 3, 4, 5). Lesions in this category were closely compared with those previously described as idiopathic oral ulcerative lesions and which had created diagnostic uncertainty during the FMD outbreak of 2001 [5, 21]. In this study, idiopathic oral lesions were most commonly ulcerative. Oral erosive/ulcerative lesions were most commonly encountered on the dental pad (10/25 40 % of lesions in this category; 2/10 from the flock study, 8/10 from the abattoir study).

Video footage of three of the idiopathic oral erosive/ulcerative lesions included in this category is presented in the Additional files 1 to 3.

**Fig. 6:**
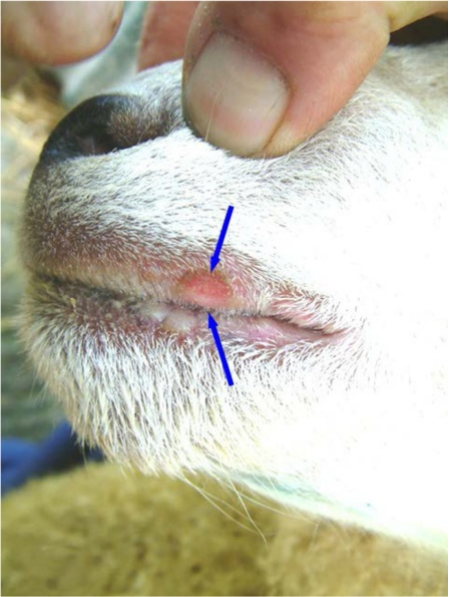
Photograph illustrating a red, semi-circular shaped erosive ulcer, approximately 5 mm in diameter on the lateral aspect of the upper lip, near the mucocutaneous junction and approximately 2 cm from the right commissure (blue arrows). This lesion was observed in a lamb from a flock where orf was endemic

**Fig. 7:**
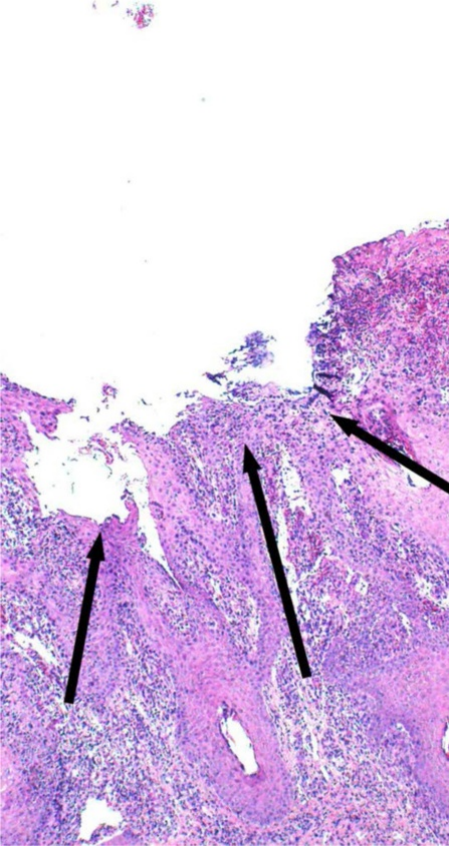
Photomicrograph illustrating a section of the ulcer shown in Fig. 6. There is extensive epidermal necrosis and hyperplasia with a dense infiltrate of inflammatory cells, areas of erosion and ulceration (black arrows) (HE 4x)

**Fig. 8:**
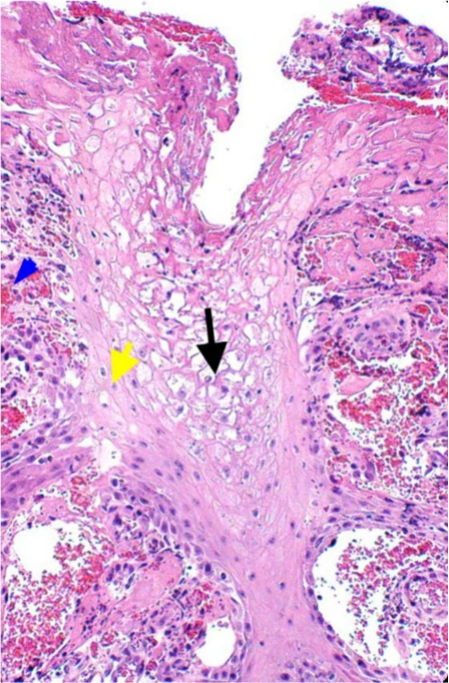
Photomicrograph illustrating another section of the ulcer depicted in Fig. 6. Highlighted areas illustrate swollen keratinocytes (yellow arrow) and reticular keratinocyte degeneration within the hyperplastic epidermis (black arrow). There is significant congestion and haemorrhage in the subjacent dermis (blue arrow heads) (HE 10x)

Orf virus was not detected from any of these lesions, whilst bacterial species isolated were considered to be commensals at this location and included: *Staphylococcus* species, *Streptococcus* species and *Mannheimia haemolytica* [22]. In one case (Figs. 6, 7, 8) there was no evidence of viral involvement on EM or immunohistochemical staining, but histopathological changes consistent with orf were present [19].

### Oral exophytic lesions

**Fig. 9:**
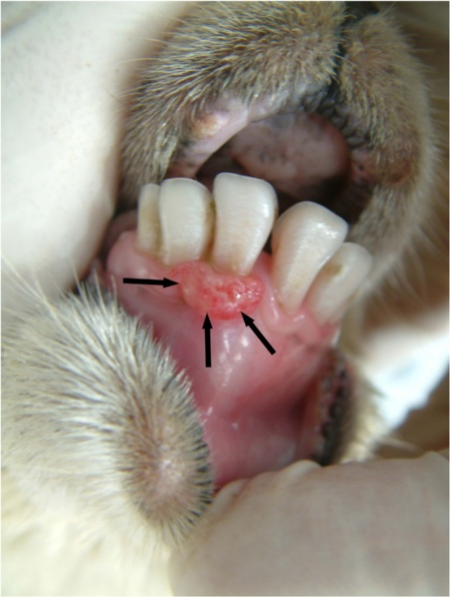
Photograph illustrating an oral exophytic lesion (black arrows). The lesion is solitary, erythematous, elliptical and slightly nodular. It is located on the maxillary mucosa adjacent to the base of the right-hand middle incisor. This lesion was confirmed as contagious pustular dermatitis (orf) by Electron Microscopy

**Fig. 10:**
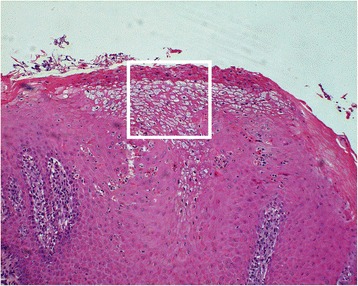
Photomicrograph illustrating a section through the lesion highlighted in Fig. 9. There is hyperkeratosis, epidermal hyperplasia and vacuolar degeneration in the superficial layers of the epithelium (H&E, 10x). The area within the white box is magnified in Fig. 11

**Fig. 11:**
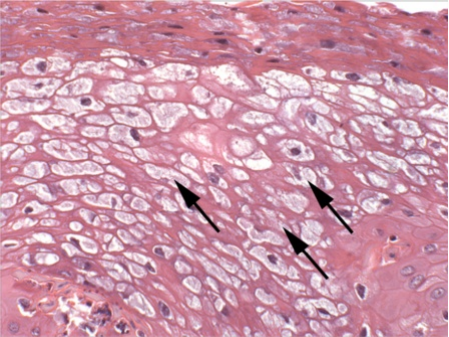
Photomicrograph of the highlighted area in Fig. 10, displaying reticular degeneration of the keratinocytes (black arrows) (HE 40x)

Exophytic lesions were defined as lesions which were growing or extending outwards from the skin/mucosal surface [20]. There were 19 lesions within this category, the majority (12/19, 63 %) of which were subsequently diagnosed as contagious pustular dermatitis or orf (Figs. 9, 10, 11). Diagnosis was based on the detection of virus by EM, a positive immunohistochemistry staining and the presence of characteristic histopathological changes. These included an irregularly hyperplastic epidermis with a serocellular crust, the presence of reticular and vacuolar keratinocyte degeneration and perivascular cuffing within the superficial dermis [23, 24]. The remaining 7 cases comprised of 6 lesions which were consistent with the gross morphological characteristics of contagious pustular dermatitis (orf), and were described in lambs within flocks with a history of orf but from which orf virus was not detected by EM or immunohistochemistry. The last lesion within this group was detected in a housed ewe, fed on silage and concentrates from wooden troughs. The ewe was not pyrexic and a definitive diagnosis was not reached as to the aetiology.

### Digital lesions

The 20 digital lesions identified in the two studies were also classified as either erosive/ulcerative or exophytic. There were 13 (65 %) and 7 (35 %) lesions in the erosive/ulcerative and exophytic categories, respectively. Diagnoses were as follows: foot-rot (6/20, 30 %), white line disease (shelly hoof) (6/20, 30 %), interdigital dermatitis (scald) (5/20, 25 %), interdigital fibroma (2/20, 10 %) and toe fibroma (1/20, 5 %). Of the 20 sheep affected, three (15 %) were lame, whilst eight (40 %) displayed evidence of localised lymph node enlargement (the popliteal lymph node (5/8, 63 %) and the prescapular lymph node (3/8, 37 %)). The most commonly isolated bacteria represented in the two categories of lesions were *Fusobacterium necrophorum* (8/20, 40 %), *Escherichia coli* (7/20, 35 %), *Peptostreptococcus indolicus* (5/20, 25 %), *Trueperella pyogenes* (3/20, 15 %) and *Bacillus licheniformis* (3/20, 15 %). No viruses were detected in any samples.

## Discussion

During the FMD outbreak in the UK and Ireland in 2001, there was considerable uncertainty surrounding the diagnosis of FMD on clinical grounds in sheep [6, 25, 26]. The present study, conducted in a FMD-free situation, aimed to address this uncertainty by systematically identifying, describing and categorising the background range of oral and digital lesions found in sheep in Ireland.

**Fig. 12:**
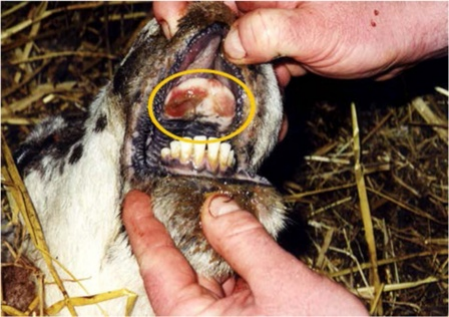
Photograph illustrating multiple ulcers (encircled) on the dental pad, a predilection site of ulceration in sheep infected with FMD, taken *post mortem* (Photograph courtesy of K. Dalzell, DARD NI)

Examination of 5,666 sheep in this survey revealed the presence of oral lesions in 0.8 % of animals. A higher lesion prevalence was found in lambs (1.3 %) compared to adult animals (0.7 %). The lesions could be divided morphologically into erosive/ulcerative and exophytic types. Evidence of orf virus was detected in the majority of the latter type of lesions while the aetiology of the erosive/ulcerative oral lesions was not determined. All of the animals with lesions were seronegative for antibodies to FMDV. There was no difficulty in differentiating these lesions from those caused by FMDV (Fig. 12) on the basis of flock history and careful clinical examination.

A number of hypotheses as to the pathogenesis of the idiopathic oral ulcerative lesions have been proposed. Direct trauma to the oral mucosa may occur due to grazing short grass on rough ground or possibly as a result of dosing gun injuries [27]. It was suggested that the gingivae could be abraded by stone- and grit-contaminated herbage [28], by the feeding of cuboidal salt blocks [29, 30], or due to browsing on emerging hawthorn (*Crataegus monogyna*) or rushes (*Juncus effusus*) [26].

Idiopathic oral ulceration has been shown to have a low prevalence ranging from 0.95 % to 1.15 % in the UK [31], whilst the prevalence of idiopathic oral lesions in New Zealand has been reported to be up to 4 % [27]. In the present study, in 1,264 adults, the prevalence was 0.7 %, and in the abattoir study it was 0.5 %. The New Zealand and UK abattoir studies on adult sheep were conducted on samples of 13,000 and 17,963 animals respectively [27, 31]. The marginally lower prevalence recorded in the present study may reflect the smaller sample size as well as variations in grazing surface conditions or dosing techniques, or possibly other unidentified factors.

Idiopathic lesions of oral ulceration were described in previous studies as solitary or multiple, red, raw ulcerated areas surrounded by a hyperaemic border on the lower gum ventral to the incisor teeth with lesions less frequently seen on the upper gum, dental pad, hard palate and tongue [6, 31]. In contrast to FMD, idiopathic oral ulcerative and erosive lesions were ulcerative not vesicular [30]. Additionally, animals with idiopathic oral ulcerative and erosive lesions were not pyretic and there was no evidence of sudden acute transient lameness, milk drop or neonatal mortality in any of these flocks; all signs typically seen in cases of FMD.

The histopathological appearance of lesions in the oral erosive/ulcerative (idiopathic) category in the present study suggested bacterial infection. The bacteria isolated were consistent with opportunistic commensal organisms [22]. Many of these ulcerative idiopathic lesions exhibited accompanying epidermal hyperplasia with heavy neutrophilic and lymphocytic infiltration at the deepest margin of the ulcer. The lesions displayed little evidence of healing, suggesting that they were recently formed. As such, the lesions resembled the ulcerated lesions, described at various stages of healing and repair in New Zealand [27] and the UK[31]. In those surveys, lesions were attributed to traumatic causes including collisions with fencing due to flight/fright reactions from people and dogs and following exposure to abrasive soil, plant material or mineral blocks.

In the UK, where these idiopathic oral ulcerative lesions were identified, foot lesions were not recorded in the same animals [5]. However, in Ireland during the FMD outbreak, sheep exhibiting lesions attributed to idiopathic oral ulceration were also found to have digital lesions [32]. However, the diagnostic significance and relevance of this finding to the diagnosis of FMD is unclear. Digital lesions associated with FMDV may not be common in some outbreaks of FMD and lesions attributable to foot-rot in FMD-affected sheep have been frequently reported [3]. In the present study, sheep with idiopathic oral ulcers did not display foot lesions.

Contagious pustular dermatitis (orf) was the most commonly diagnosed cause of oral lesions in the present study, confirmed in 12 out of 44 cases (27 %) i.e. 12 out of 19 oral exophytic lesions (63 %). Whilst the prevalence of orf amongst lambs in Irish flocks is unknown, studies establishing prevalence of orf in New Zealand (4.1 %) [33] and in Turkey (52.8 %) [34] have been published. The findings of this study indicate that orf infection is common in Irish flocks. However, as the flocks examined in the flock study and the abattoirs visited during the abattoir study, were not selected randomly, the prevalence of orf recorded may not accurately reflect the incidence of the disease nationally.

The important differential diagnoses, in the oral region, in Ireland, for FMD include contagious pustular dermatitis, idiopathic oral erosion or ulceration, junctional epidermolysis bullosa and the exotic diseases bluetongue and vesicular stomatitis. The differential diagnoses for FMD in the foot region include contagious pustular dermatitis, foot-rot, contagious ovine digital dermatitis, junctional epidermolysis bullosa and the exotic disease bluetongue.

The presence of pyrexia with sudden onset severe lameness characterised by vesicle formation tends to eliminate other common causes of foot lameness such as virulent foot-rot [35]. However, it is noteworthy that pyrexia is not uncommonly detected in sheep that have been recently gathered and during the 2001 epidemic several flocks were falsely diagnosed as having FMD on the basis of pyrexia alone [13, 35].

The relevant differential diagnoses of FMD in sheep include the exotic disease of bluetongue infection (BT). Signs of BT include initial pyrexia [36, 37] leading to depression and nasal discharge which may be serous, mucopurulent or bloody. Hyperaemia of the oral mucosa, a common presenting clinical sign, may be accompanied by mucosal ulceration or erosion [4]. There may be drooling of saliva (ptyalism) due to oral lesions and tongue involvement, facial oedema and muscle weakness. In severe cases, the tongue may be so badly affected that the epithelial surface may be stripped from it. The tongue then becomes very oedematous and swollen, making eating very difficult [38].

## Conclusion

In conclusion, whilst the lesions profiled in the present study bore little resemblance to the classical oral lesions described in cases of FMD in sheep, the presence of idiopathic oral ulcers should be considered in the context of the differential diagnosis of FMD in the field.

## Additional files

Additional file 1:
**Clip 1.** Idiopathic Lesion 1: This lesion was seen in a hogget ewe, which was grazing on root crops. It highlights an example of an oral erosive/ulcerative lesion as categorised in our study. It is an ulcer, which is approximately 1 cm in diameter located on the mandibular gum. It is located roughly along the midline and is approximately 1 to 1.5 cm ventral to the teeth. The ulcer has a pale to white circular border surrounding a triangular red centre. This animal was subjected to a full clinical examination prior to capturing this footage and all values were within normal ranges. There was no evidence of localised or generalised lymphadenopathy. (MPEG 7.04 mb) Additional file 2:
**Clip 2.** Idiopathic Lesion 2: This lesion was seen in a broken mouthed ewe in the same flock as the sheep in clip number 1. The ewe was grazing on root crops. The lesion also highlights an example of an oral erosive/ulcerative lesion. It is an elliptical ulcer approx 1 cm long by 0.5 cm wide. It is located on the lingual aspect of the lower lip. Detailed microbiological examination did not reveal any aetiology. This combined with the location of the lesion and the grazing history suggests that this lesion was likely traumatic in origin. (MPEG 12.1 mb) Additional file 3:
**Clip 3.** Lesion 3: This exophytic lesion was seen in a young lamb in a flock with a history of contagious pustular dermatitis (orf). There are exophytic rough textured lesions on the maxillary (either side of the philtrum approximately 2-3 cm in diameter) and mandibular (on the rostral surface along the midline, approximately 3-4 cm in diameter) lips of this lamb. On the surface of these lesions, there is yellowish material. When the mouth of the lamb is opened, there are 2 target shaped vesicular lesions on the rostral aspect of the dental pad, approximately 1 cm in diameter, located approximately 1 cm lateral to the midline. Upon investigation of these lesions, they were confirmed to be orf on electron microscopy. (M4V 10.3 mb)

## Abbreviations

CODD: Contagious ovine digital dermatitis
EM: Electron microscopy
ELISA: Enzyme linked immunosorbent assay
FMD: Foot-and-mouth disease
FMDV: Foot-and-mouth disease virus
HE: Haematoxylin and Eosin
IAH: Institute for animal health
LPB-ELISA: Liquid phase blocking enzyme linked immunosorbent assay
UK: United Kingdom
